# Research on prediction of compressive strength of fly ash and slag mixed concrete based on machine learning

**DOI:** 10.1371/journal.pone.0279293

**Published:** 2022-12-27

**Authors:** Meng Wang, Jiaxu Kang, Weiwei Liu, Jinshuai Su, Meng Li

**Affiliations:** College of Mining Engineering, Liaoning Technical University, Fuxin, China; Instituto Federal do Espírito Santo: Instituto Federal de Educacao Ciencia e Tecnologia do Espirito Santo, BRAZIL

## Abstract

Every year, a large amount of solid waste such as fly ash and slag is generated worldwide. If these solid wastes are used in concrete mixes to make concrete, it can effectively save resources and protect the environment. The compressive strength of concrete is an essential indicator for testing its quality, and its prediction is affected by many factors. It is difficult to predict its strength accurately. Therefore, based on the current popular machine learning supervised learning algorithms: Random Forest (RF), Extreme Gradient Boosting (XGBoost), and Support Vector Machine (SVR), three models established a nonlinear mapping between multi-factor features and target feature concrete compressive strength. Using the three completed training models, we validated the test set with 206 example sets, and the Root Mean Square Error (RMSE), fitting coefficient (R^2^), and Mean Absolute Error (MAE) were used as evaluation metrics. The validation results showed that the values of RMSE, R^2^, and MAE for the RF model were 0.1, 0.9, and 0.21, respectively; the values of XGBoost model were 0.05, 0.95, and 0.15, respectively. The values of SVR were 0.15, 0.86, and 0.3, respectively. As a result, Extreme Gradient Boosting (XGBoost) has better generalization ability and prediction accuracy than the other two algorithms.

## 1. Introduction

Concrete is widely used in the construction industry because of its excellent performance [1]. Every year the whole world, because of the construction needs, the amount of use of concrete will be huge [2]. Thus, the carbon footprint associated with cement, and therefore conventional concrete, production is high. Increasing the use of supplementary cementitious materials (SCMs) in concrete is an obvious and necessary step to reduce carbon emissions [3, 4]. Mohamed and Tayeb [5] have studied that concrete is generally mixed with cement, sand, stone, water, and other materials by a certain proportion of the formation of a rigid material. SCMs, such as fly ash and slag are often waste materials from industrial processes. The wide application of these solid wastes in concrete has become an effective measure to reduce the cement consumption and delay the hydration heat, its application not only has a good economy but also can make the concrete have better mixing performance, hardening properties, and durable performance [6–8]. Inclusion of SCMs in concrete lessens the environmental impact of concrete in several ways, in that it: (1) reduces cement consumption and thereby production, (2) can reduce the amount of inert filler (typically sand in conventional concrete) required and (3) uses waste materials that would otherwise be landfilled.

In the current studies, the machine learning-based model has been widely used in slope stability prediction [2], floods [9], prediction of mechanical properties of materials [10–12] and building structures [13, 14]. In addition, many researchers have explored the application of machine learning in concrete prediction of compressive strengths. For example, the studies of Yeh et al. [15–17], built an ANN model with a backpropagation algorithm to predict the compressive strength of concrete at different ages (3 days, 14 days, 28 days, and 90 days). Fan and Chiong et al. [18] predicted the compressive strength of concrete with a support vector machine by studying the composition of concrete. The experimental results show that the model has good performance for the reverse prediction of concrete members under the conditions of multiple input, single output and multiple input and multiple output. Behnood and Golafshani [19] used decision trees to predict the compressive strength of concrete with fly ash and other waste materials; The results indicated that the proposed models could provide reliable predictions of the target mechanical properties. Deshpande and Londhe [20] estimated the compressive strength of recycled aggregate concrete with the artificial meridian; The results indicate that ANN learns from the examples and grasped the fundamental domain rules governing strength of concrete. At present, the research on making concrete with solid wastes such as fly ash and slag as mixing material needs to be further improved [21]. Because of the small sample size of previous studies, the over-fitting problem is easy to occur in model training. The data set samples used in this study reached 1030 groups, which has a better optimization for the over-fitting problem. Using limited data to predict the compressive strength of this type of concrete will help realize engineering applications. At the same time, compared with previous models, the application of a more advanced XGBoost model to a concrete compressive strength test is also helpful to improve the prediction accuracy and speed; In previous experiments also have the problem of lack of field experiment data validation, theoretical research should return to the practical application, most of these studies only compared to the existing data sets, without actually making a concrete test block, a lack of practicality, to solve this problem, after completing the model performance comparison, concrete test blocks will be made on-site. The compressive strength test is carried out after curing for a fixed time in the constant temperature curing box to verify the model’s accuracy.

## 2. Research methods and steps

The mechanical properties of concrete under the action of multi-feature coupling are affected by many factors, which overlap and influence each other, showing a very complex nonlinear relationship [22]. In this paper, the prediction accuracy and practicability of the three machine learning models, XGBoost model, SVR model, and RF model, will be evaluated by comparing the curve fitting degree between the predicted value and the actual value of the three machine learning models, as well as statistical indicators such as RMSE, R^2^, and MAE, as well as experimental verification. The programming language used was Python3.9, programming analysis on the Notebook platform.

**Fig 1:**
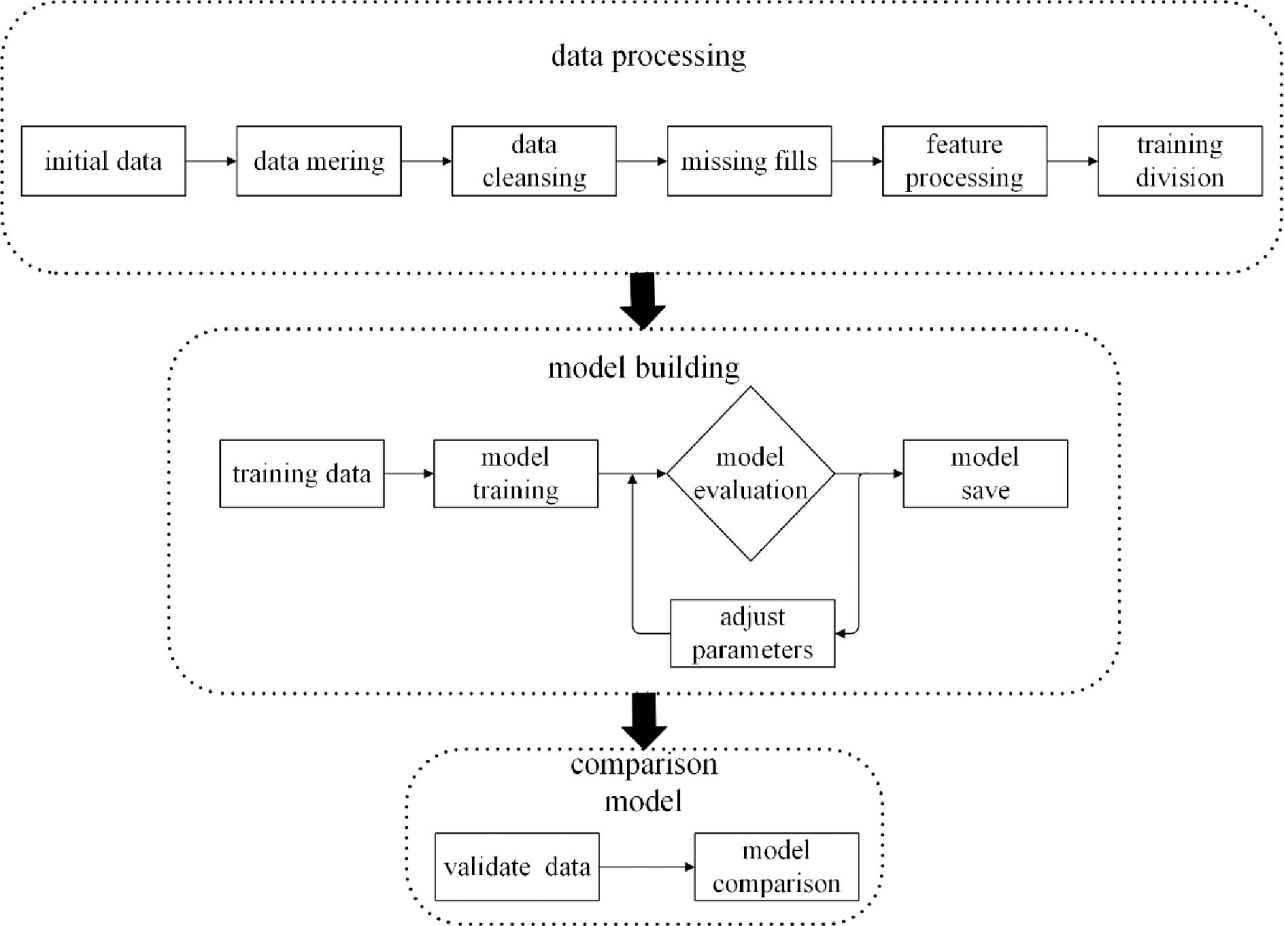
Model training and experimental verification process.

To better conduct model training and model verification, this study will follow the ideas shown in Fig 1, mainly including data processing, model training, model testing, model comparison, and experimental validation. The specific steps are as follows:

1. Data preprocessing, the data were first preprocessed, such as quality control and dataset partitioning, and correlation analysis.
2. In machine learning model construction, first build three models of RF, XGBoost, and SVR. After obtaining the initial model, use the training set samples to train the model, then use the divided test set samples to test the three models, and optimize through grid search parameters, so that the model performance is optimal. Finally, the model with the optimal parameters is selected [23].
3. Comparison of model prediction performance. By inputting the test set into three models and obtaining the model predictive value, draw the fitting curve, scatter chart, relative error chart and Taylor chart between the actual value and the predictive value, and calculate Root Mean Square Error (RMSE), coefficient (R^2^), Mean Absolute Error (MAE). Then the prediction performance of the model is evaluated according to the graph and statistical indicators, and the model with the best comprehensive performance is obtained [24].
4. Example verification of optimal model. Make a concrete test block with the same characteristics as the data set, put the completed test block into a constant temperature curing box for 28 days, perform a compressive strength test on the cured concrete test block and record the experimental data; The values of the parameters are input into the optimal model, and the compressive strength is predicted; the average error rate is calculated by comparing the experimental data and the expected data, to test the performance of the model in the actual working state [25].

## 3. Data analysis

### 3.1 Sample data acquisition and preprocessing

The sample data were based on the Concrete Compressive Strength dataset [26], provided by Professor Yicheng Ye, Department of Information Management, Chunghua University, Taiwan [27] (Due to the large amount of data can not be fully displayed, only part of the data is shown here, the complete data is detailed in this data warehouse: https://doi.org/10.6084/m9.figshare.21493236.v1). The sample dataset consisted of 1030 sets of samples. Each of these had nine features: cement, blast furnace slag, fly ash, water, superplasticizer, coarse aggregate, fine aggregate, age, and concrete compressive strength. The first eight features are input features, and the ninth feature is the target feature. Eight hundred twenty-four groups of samples are randomly assigned as training samples, and the remaining 206 groups are test samples. The specific mix proportion and strength test values of some samples are shown in Table 1; The relevant statistical description of the data set is shown in Table 2.

**Table 1:** Experimental data.

ordinal	cement (kg/m^3^)	blast furnace slag (kg/m^3^)	fly ash (kg/m^3^)	water (kg /m^3^)	superplasticizer (kg /m^3^)	coarse aggregate (kg /m^3^)	fine aggregate (kg /m^3^)	age (day)	compressive strength (MPa)
1	500.0	0.0	0.0	200.0	0.0	1125	613.0	1	12.64
2	385.0	0.0	0.0	186.0	0.0	966	763.0	1	6.27
3	139.6	209.4	0.0	192.0	0.0	1047	806.9	3	8.06
4	349.0	0.0	0.0	192.0	0.0	1047	806.9	3	15.05
5	198.6	132.4	0.0	192.0	0.0	978.4	825.5	3	9.13
6	310.0	0.0	0.0	192.0	0.0	971.0	850.6	3	9.87
7	374.0	189.2	0.0	170.1	10.1	926.1	756.7	3	34.40
8	313.3	262.2	0.0	175.5	8.6	1047	611.8	3	28.80
9	425.0	106.3	0.0	153.5	16.5	852.1	887.1	3	33.40
10	425.0	106.3	0.0	151.4	18.6	936.0	803.7	3	36.30
11	375.0	93.8	0.0	126.6	23.4	852.1	992.6	3	29.00
12	475.0	118.8	0.0	181.1	8.9	852.1	781.5	3	37.80
13	469.0	117.2	0.0	137.8	32.2	852.1	840.5	3	40.20
14	425.0	106.3	0.0	153.5	16.5	852.1	887.1	3	33.40
~	~	~	~	~	~	~	~	~	~
1025	237.5	237.5	0.0	228.0	0.0	932.0	594.0	365	39.00
1026	475.0	0.0	0.0	228.0	0.0	932.0	594.0	365	41.93
1027	339.0	0.0	0.0	197.0	0.0	968.0	781.0	365	38.89
1028	236.0	0.0	0.0	193.0	0.0	968.0	885.0	365	25.08
1029	254.0	0.0	0.0	198.0	0.0	968.0	863.0	365	29.79
1030	307	0.0	0.0	193.0	0.0	968.0	812.0	365	36.15

**Table 2:** Overall statistical description of data.

	cement (kg/m^3^)	blast furnace slag (kg /m^3^)	fly ash (kg /m^3^)	water (kg /m^3^)	superplasticizer (kg /m^3^)	coarse aggregate (kg /m^3^)	fine aggregate (kg /m^3^)	age (day)	compressive strength (MPa)
count	1030	1030	1030	1030	1030	1030	1030	1030	1030
mean	281.17	73.90	54.19	181.57	6.20	972.92	773.58	45.66	35.82
std	104.51	86.28	64.00	21.36	5.97	77.75	80.18	63.17	16.71
min	102.00	0.00	0.00	121.75	0.00	801.00	594.00	1.00	2.33
max	540.00	359.40	200.10	247.00	32.20	1145.0	992.60	365.0	82.60

### 3.2 Correlation analysis of sample data

**Fig 2:**
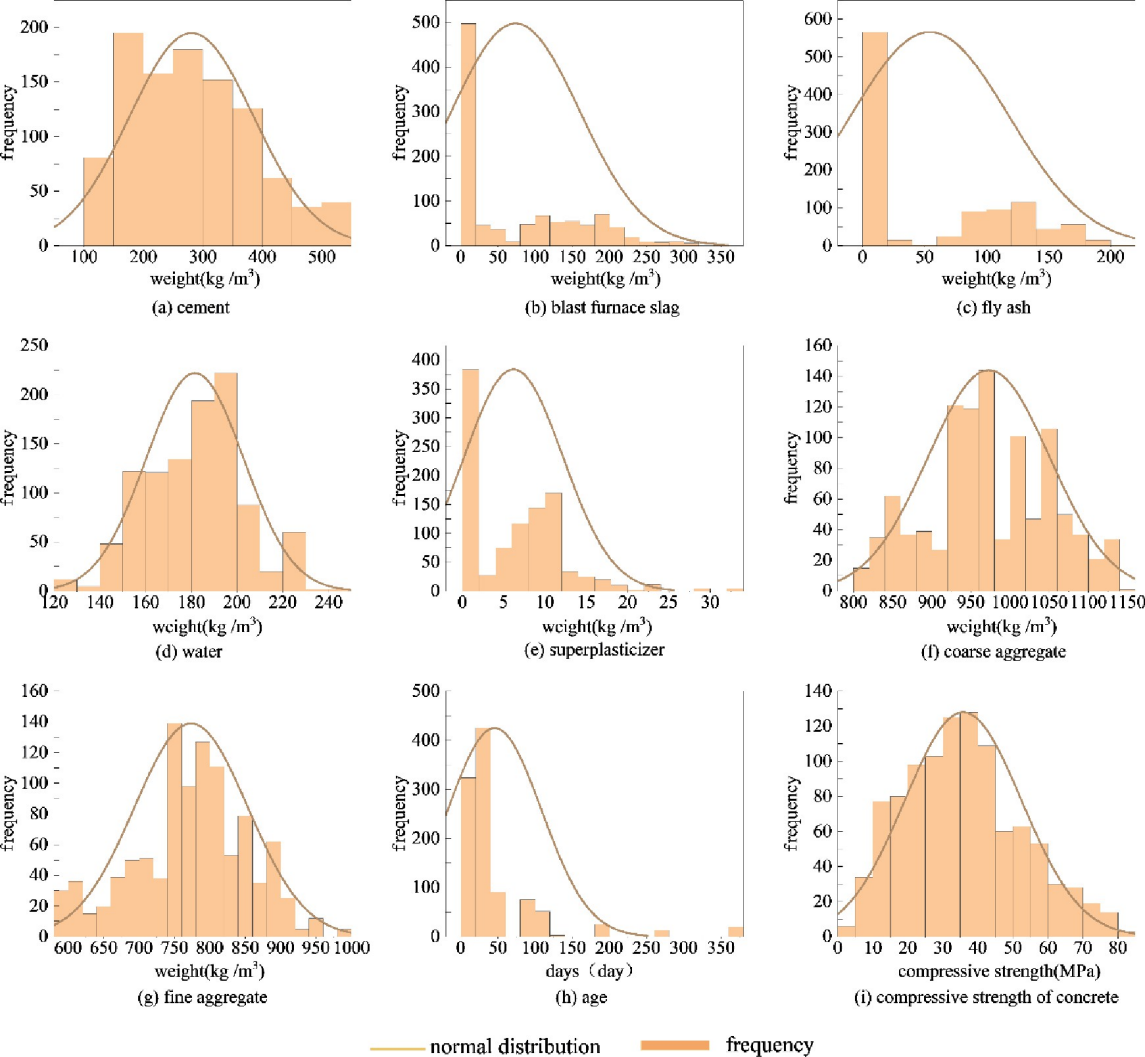
Characteristic numerical distribution. (a) cement; (b) blast furnace slag; (c) fly ash; (d) water; (e) superplasticizer; (f) coarse aggregate; (g) fine aggregate; (h) age; (i) compressive strength of concrete.

Before model training, samples’ features unrelated to concrete compressive strength should be excluded, so correlation analysis should be conducted. Correlation analysis needs to verify the distribution state of sample data; the histogram and normal curve of each characteristic value distribution is shown in Fig 2. There are three commonly used correlation coefficients: Pearson correlation coefficient, Spearman correlation coefficient, and Kendall correlation coefficient. The two variables using the Pearson correlation coefficient should follow a normal distribution and have a linear correlation trend; the Pearson correlation coefficient is used to measure the degree of linear correlation [28]. The value range is [–1,1]. The larger the absolute value of the coefficient is, the stronger the correlation is; the closer the absolute value is to 0, the weaker the correlation is. As shown in Fig 2, Fig 2(A), 2(D), 2(F) and 2(G) follow a normal distribution with Fig 2(I), so the Pearson correlation coefficient is used to determine their correlation. The Pearson correlation coefficient is defined as follows:

$$\rho_{XY}=\frac{Cov(X,Y)}{\sqrt{D(X)}\sqrt{D(Y)}}=\frac{E(X-EX)E(Y-EY)}{\sqrt{D(X)}\sqrt{D(Y)}}$$ (1)

In the Eq (1): *E* is the mathematical expectation; *D* is the variance; $\sqrt{D}$ is the standard deviation; *Cov(X*,*Y)* is the covariance of the sum of the random variables, which measures the overall error between the two variables; *ρ*_*XY*_ is the value of the quotient between the covariance and standard deviation between the two variables, also known as the correlation coefficient between the variables *X* and *Y*. Variables *X* and *Y* in Eq (1) refer to two independent bodies of evidence [29].

If two variables do not obey normal distribution, the spearman correlation coefficient should be used to study their relationship. In Fig 2(B), 2(C), 2(E), 2(H) and 2(I) does not obey the normal distribution, so the Spearman correlation coefficient is used as the judgment basis of correlation. The Spielman correlation coefficient calculation expression is as follows [30, 31]:

$$\rho =1-\frac{6\sum D^{2}}{N(N^{2}-1)}$$ (2)

In the Eq (2): *ρ* is the Spearman correlation coefficient, between -1 and 1, *|ρ|* more close to 1, the greater the relevance; *D* is the difference between the two data sequences; *N* is the number of data.

**Fig 3:**
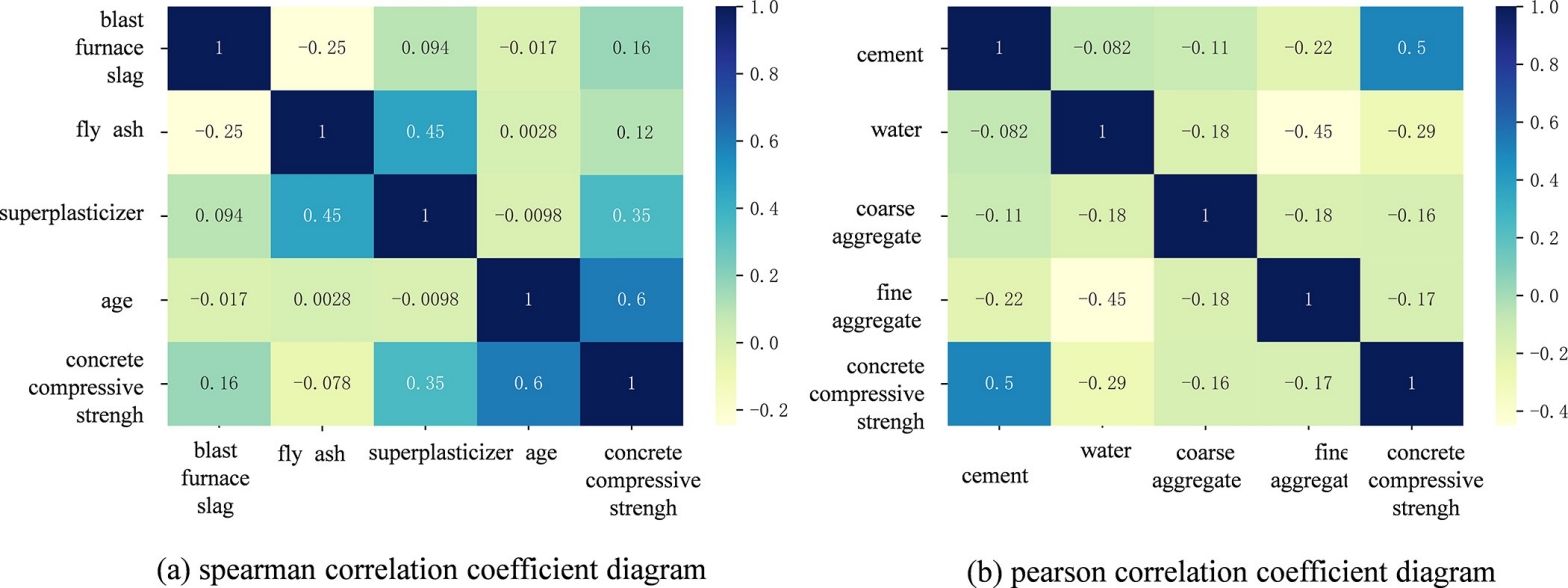
Correlation between features. (a) Spearman correlation coefficient diagram; (b) Pearson correlation coefficient diagram.

The correlation diagram calculated by Pearson correlation coefficient and Spearman correlation coefficient is shown in Fig 3. In Fig 3, Fig 3(A) is calculated from the Spearman correlation coefficient, and The Pearson correlation coefficient calculates Fig 3(B). It can be seen that the compressive strength of concrete is positively correlated with cement, blast furnace slag, superplasticizer, and age. The compressive strength of concrete is negatively correlated with coarse aggregate, fine aggregate, fly ash, and water. Among them, the compressive strength of concrete is more correlated with cement, water, superplasticizer, and age, and there are no unrelated features. The correlation diagram of each feature is realized by the Heatmap function of the Seaborn library in Python.

### 3.3 Normalization processing

There are nine features in the selected dataset, and the dimensions of the nine features are different. In order to improve the accuracy and stability of the model, reduce the amount of calculation, and achieve the best prediction effect, it is necessary to normalize the data in the dataset. Because the dataset is already fixed and new samples will not continue to be added, the maximum and minimum values of each feature will not change, so the normalization processing in this article uses the max-min mapping function, which is implemented with the help of the StandardScaler function under the sklearn library in Python, and the function form is as follows [32]:

$$X^{*}=\frac{x-\min}{\max-\min}$$ (3)

In the Eq (3): X^*^ is the mapped numeric value; *x* represents the value in the original data set; min is the minimum value of each feature; max is the maximum value for each feature.

## 4. Model introduction and model training

### 4.1 Extreme gradient boosting (XGBoost)

The extreme Gradient Boosting (XGBoost) model is based on the tree model, which is an improved model based on Gradient Boosting Decision Tree (GBDT). It is an addition formula composed of k base models proposed by Chen Tianqi, a scholar at the University of Washington. The basic idea is to use the new base model to fit the deviation of the previous model to continuously reduce the variation of the additive model [33]. By introducing regularization to the objective function and using the second derivative approximation, the XGBoost model effectively reduces the overfitting problem and improves the computational efficiency of the algorithm. Compared with other machine learning algorithms, the XGBoost algorithm is faster and more generalized. The prediction model formula is as follows:

$$\hat{y_{i}}=\sum_{i=1}^{k}f_{t}(x_{i})$$ (4)

In the Eq (4): *f*_t_ is the kth base model; $\hat{y_{i}}$ is the predicted value of the ith sample.

To avoid overfitting, the regularization term on a single base model is as follows:

$$\Omega (f)=\gamma T+\frac{1}{2}\lambda {\left\|\omega \right\|}^{2}=\gamma T+\frac{1}{2}\lambda \sum_{j=1}^{T}\omega_{j}^{2}$$ (5)

In the Eq (5): *T* is the leaf node of the tree; *ω* is the output value of *x* falling on a leaf node; γ and λ are non-negative coefficients; *ω*_*j*_ is the output value of the *j*_*th*_ node.

Therefore, the XGBoost loss function is defined as:

$$Loss=\sum_{i=1}^{n}l(y_{i},\hat{y_{i}})+\sum_{k}^{}\Omega (f_{k})$$ (6)

The minimum value of the loss function is obtained using Taylor’s second-order expansion, and then the segmentation point with the highest score is searched by exact or approximate methods, and the next step is to segment and expand the leaf nodes [34].

### 4.2 Random Forest (RF)

The Random Forest (RF) model is based on the decision tree model under the bagging framework. The bagging with the decision tree as the primary model generates a decision tree after each bootstrap is put back into the sampling, there is no further intervention in developing these trees; random forest also performs bootstrap sampling, but the difference between it and bagging is that when generating each tree, each node variable is only generated in a few randomly selected variables, Therefore, not only the samples are random, but also the generation of each node variable is random; The advantage is that it can transform a limited number of weak classifiers into a strong classifier through linear combination, to improve the accuracy and robustness [35]. The problem studied in this paper is a regression problem. The random forest regression algorithm outputs the results of all decision trees and then takes the mean value. The regression equation is as follows:

$$\bar{H}(x)=\frac{1}{K}\sum_{i=1}^{k}{\left(h_{i}(x,\theta_{k})\right)}^{}$$ (7)

In the Eq (7): $\bar{H}(x)$ is the prediction result; hi is a single decision tree; θ_k_ is an independent distributed random variable that determines the growth process of a single decision tree; K is the number of decision trees.

Each decision tree in a random forest does not capture all features simultaneously, so each decision tree has uncertainty, so the model’s generalization ability is increased.

### 4.3 Support Vector Regression (SVR)

The Support Vector Machine (SVM) model is a data mining model based on statistical theory, and its purpose is to find an optimal hyperplane to separate two different classes. When Support Vector Regression is used for regression problems, the model is called Support Vector Regression(SVR); when dealing with SVR nonlinear problems, the kernel function maps nonlinear problems in a low-dimensional space to a high-dimensional feature space, and finds the optimal hyperplane in the high-dimensional space, and then calculate the distance between all training set samples and this plane, so that the distance is the smallest, to achieve regression prediction [36, 37]. In this paper, through multi-parameter verification, the kernel function used is RBF (Radial Basis Kernel Function), and the kernel function expression is as follows:

$$K(X,X_{i})=\exp(-\frac{{\left\|X-X_{i}\right\|}^{2}}{2\sigma })$$ (8)

In the Eq (8): σ is the width parameter of the RBF; exp is the exponential function with the natural constant e as the base; *X-X*_*i*_ is the distance between the selected center points.

High-dimensional feature space decision function:

$$f(x)=W^{T}\varphi (x)+b$$ (9)

In the Eq (9): *W* is an adjustable weight vector; *φ(x)* is a nonlinear mapping function; *b* is a constant.

Ignoring the fitting error smaller than *ε*, the SVR model can be transformed into a constrained optimization problem as follows.


$$\left\{ \begin{array}{l} \underset{W,b,\xi_{i},\xi^{*}}{\min}\frac{1}{2}W^{T}W+\frac{1}{2}c\sum_{i=1}^{l}{\left(\xi_{i}+\xi_{i}^{*}\right)}^{}\\ \left\{ \begin{array}{l} y_{i}-W^{T}\varphi (x_{i})-b\leq \varepsilon +\xi_{i}\\ -y_{i}+W^{T}\varphi (x_{i})+b\leq \varepsilon +\xi_{i}^{*}\\ \xi_{i},\xi^{*}{}_{i}\geq 0,i=1,2,\cdots ,l \end{array}\right. \end{array}\right.$$ (10)

In the Eq (10): *c* is the penalty factor; $\xi_{i}、\xi_{i}^{*}$ are a pair of relaxation factors.

By introducing the Lagrange function, the optimization problem of Eq (10) is transformed into a dual form, and the SVR objective equation is obtained through the transformation and solution as shown in Eq (11):

$$f(X)=\sum_{i=1}^{l}\left(a_{i}^{*}-a_{i})K(X_{i},X\right)+b$$ (11)

In the Eq (11): a* and *a* are dual variables; *K(X*_*i*_,*X*_*j*_*)* = *φ*(x_i_)T*φ*(x_j_)。

### 4.4 Model parameter selection

The optimal parameters of the three models are found by grid search [38]. Grid search is a model parameter optimization technique that performs an exhaustive search for the specified parameter values. First, the Cartesian product of the given parameter values is performed to obtain a combination of a finite set of parameters. Each set of parameters is used to train the model, and select a set of parameters with the best performance as the optimal parameters. The model is written in Python language, and the grid search method uses the GridSearchCV function under the sklearn library. The optimal parameters of the three models are shown in Table 3.

**Table 3:** Optimal parameters of each model.

XGBoost		RF		SVR	
n_estimators	900	n_estimators	200	kernel	rbf
Learning rate	0.3	max_depth	36	C	41
max_depth	3	min_samples_split	2	gamma	0.05
objective	reg:squarederror	min_samples_leaf	1	epsilon	0.2
booster	gbtree				

## 5. Model prediction results and performance analysis

**Fig 4:**
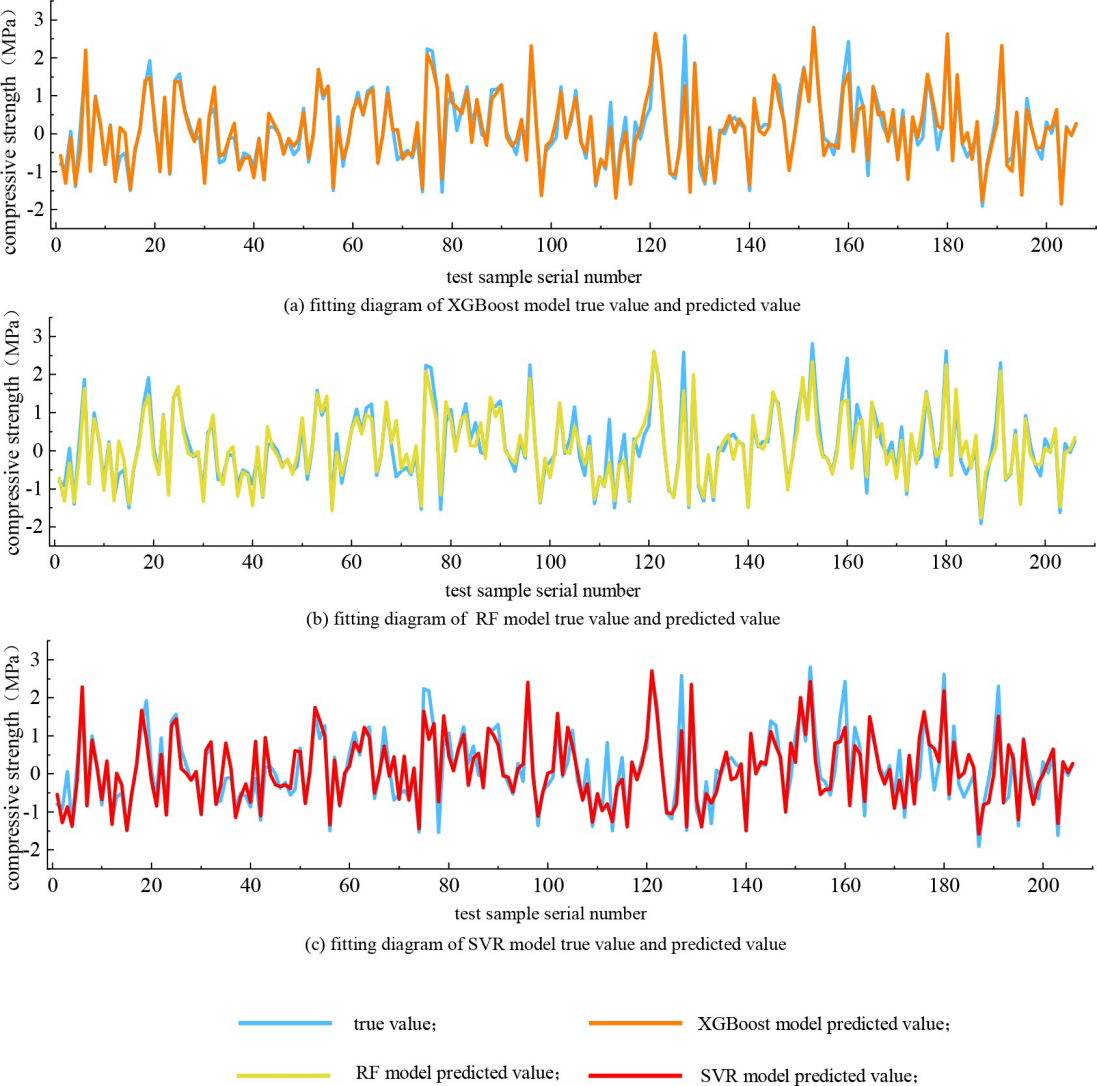
Model fitting diagram. (a) fitting diagram of XGBoost model true value and predicted value; (b) fitting diagram of RF model true value and predicted value; (c) fitting diagram of SVR model true value and predicted value.

The trained model is applied to the fitting of the verification set, and the fitting graph of the actual value and the predicted value is obtained as shown in Fig 4. The fitting diagram of the XGBoost model is shown in a in Fig 4, the fitting diagram of the RF model is shown in b in Fig 4, and the fitting diagram of the SVR model is shown in c in Fig 4. It can be seen from the Fig 4(A) that the XGBoost model has higher prediction accuracy than the other two models.

In order to evaluate the comprehensive performance of the model more objectively and combined with the fact that the problem in this study is a regression in nature, the Root Mean Square Error (RMSE), fitting coefficient (R^2^) and Mean Absolute Error (MAE) are adopted to evaluate indicators. The Root Mean Square Error (RMSE) is used to measure the degree of data change. The smaller the RMSE value is, the higher the accuracy is. The fitting coefficient R^2^ is used to measure the fitting degree between the predicted value and the real value. The closer it is to 1, the better the model fitting effect is. MAE is the sum of the absolute value difference between the real value and the predicted value. The closer it is to 0, the better the model performance is. The evaluation indices of the three models are shown in Table 4. The XGBoost model has the highest R^2^ value and the lowest RMSE and MAE value, so it performs best. At the same time, the RF model has the second highest R^2^ value and the second lowest RMSE and MAE values, which are lower than the XGBoost model in performance, and the SVR model performs poorly. The performance of traditional SVR and RF models is lower than XGBoost models. The three parameter formulas are as follows [39]:

$$RMSE=\sqrt{\frac{1}{n}\sum_{i=1}^{n}{\left(b_{ti}-b_{pi}\right)}^{2}}$$ (12)

$$R^{2}=\frac{{\left(\sum_{i=1}^{n}\left(b_{ti}-{\bar{b}}_{t}\right)\cdot (b_{pi}-{\bar{b}}_{p})\right)}^{2}}{\sum_{i=1}^{n}{\left(b_{ti}-{\bar{b}}_{t}\right)}^{2}\cdot \sum_{i=1}^{n}{\left(b_{pi}-{\bar{b}}_{p}\right)}^{2}}$$ (13)

$$MAE=\frac{1}{n}\sum_{i=1}^{n}\left|\left(b_{ti}-b_{pi}\right)\right|$$ (14)

**Table 4:** Performance index of each model.

model name	performance parameters		
	RMSE	R^2^	MAE
RF	0.1	0.9	0.21
XGBoost	0.05	0.95	0.15
SVR	0.15	0.86	0.3

In the Eqs (12)–(14): *n* is the number of data; *b*_*ti*_ is the actual value of the t feature of the *i*_*th*_ sample; *b*_*pi*_ is the predicted value of the p feature of the ith sample, $\bar{b_{t}}$ and $\bar{b_{p}}$ are the average of the actual and predicted results, respectively.

**Fig 5:**
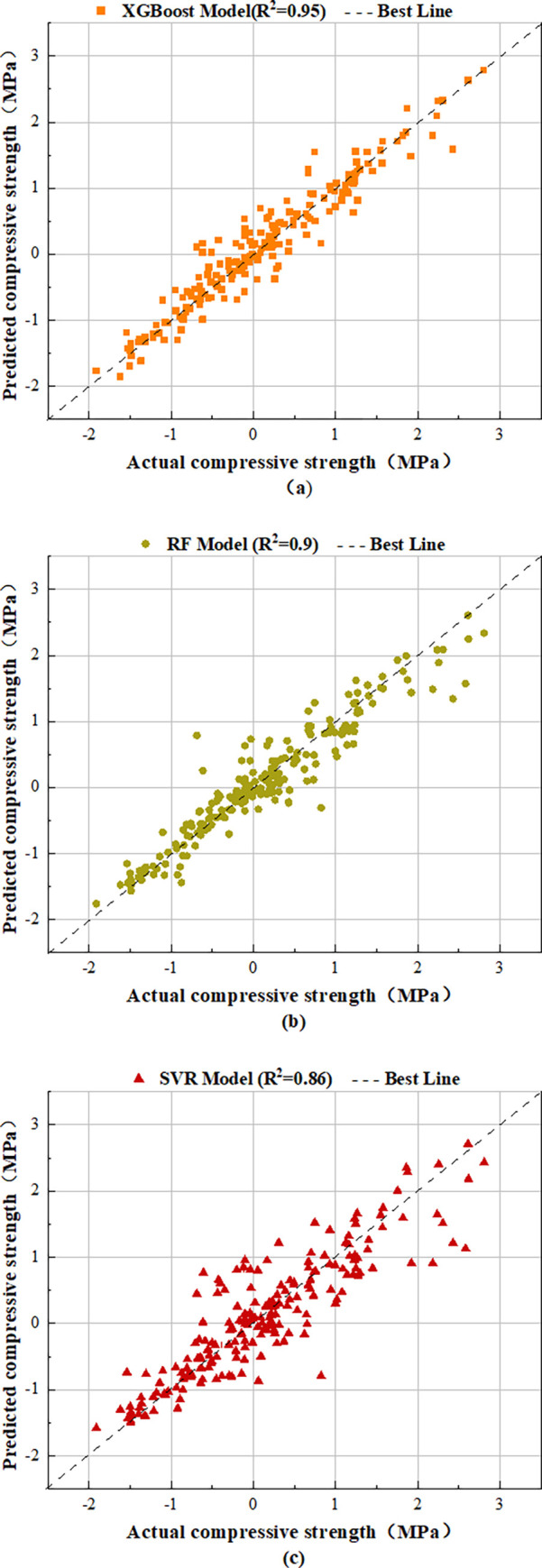
Scatter plots demonstration between actual compressive strength and predicted values obtained from machine learning models.

The applied predictive models were assessed using the feasibility of correlation analysis. Scatter plot is one of the informative graphical presentations for examining the aptitude of prediction models [40, 41]. Based on the exhibited results in Fig 5, Wherein, Fig 5(A) shows the scatter plot of the prediction results of XGBoost model, Fig 5(B) shows the scatter plot of the prediction results of RF model, and Fig 5(C) shows the scatter plot of the prediction results of SVR model. Comparing the three figures, it is found that XGBoost model can well mine the relationship between input and output after training, and the correlation between the prediction results and experimental results is closer to 1 compared with RF model and SVR model.

**Fig 6:**
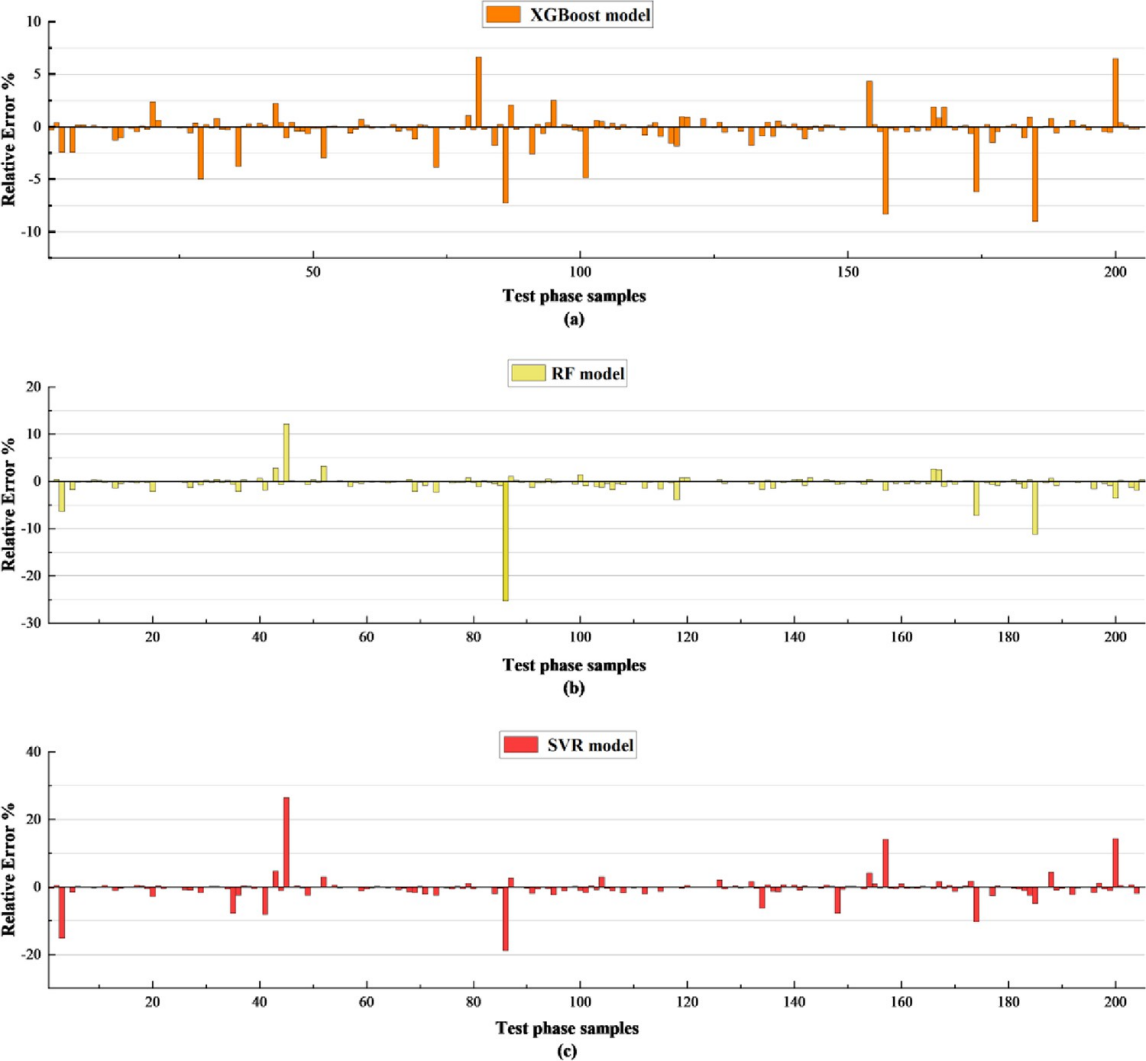
Relative error-index over the test phase sample for predicted values utilizing machine learning models.

In the testing phase, a total of 206 samples were tested, and the relative error (RE) percentage was calculated for each sample, and the RE value can visually observe the stability of the prediction model and the error extremes, as shown in Fig 6, Fig 6(A) is the relative error percentage of the XGBoost model, Fig 6(B) is the relative error percentage of the RF model, Fig 6(C) is the relative error percentage of the SVR model. The XGBoost model is more stable, with the absolute value of the error percentage within 10%, while the absolute value of the error percentage of the RF and SVR models is within 27%; in terms of the error extremes, the absolute value of the XGBoost model is 8%, while the RF model reaches 26% and the SVR model reaches 27%, based on the RE% evaluation, the XGBoost model is more stable in prediction performance [42].

**Fig 7:**
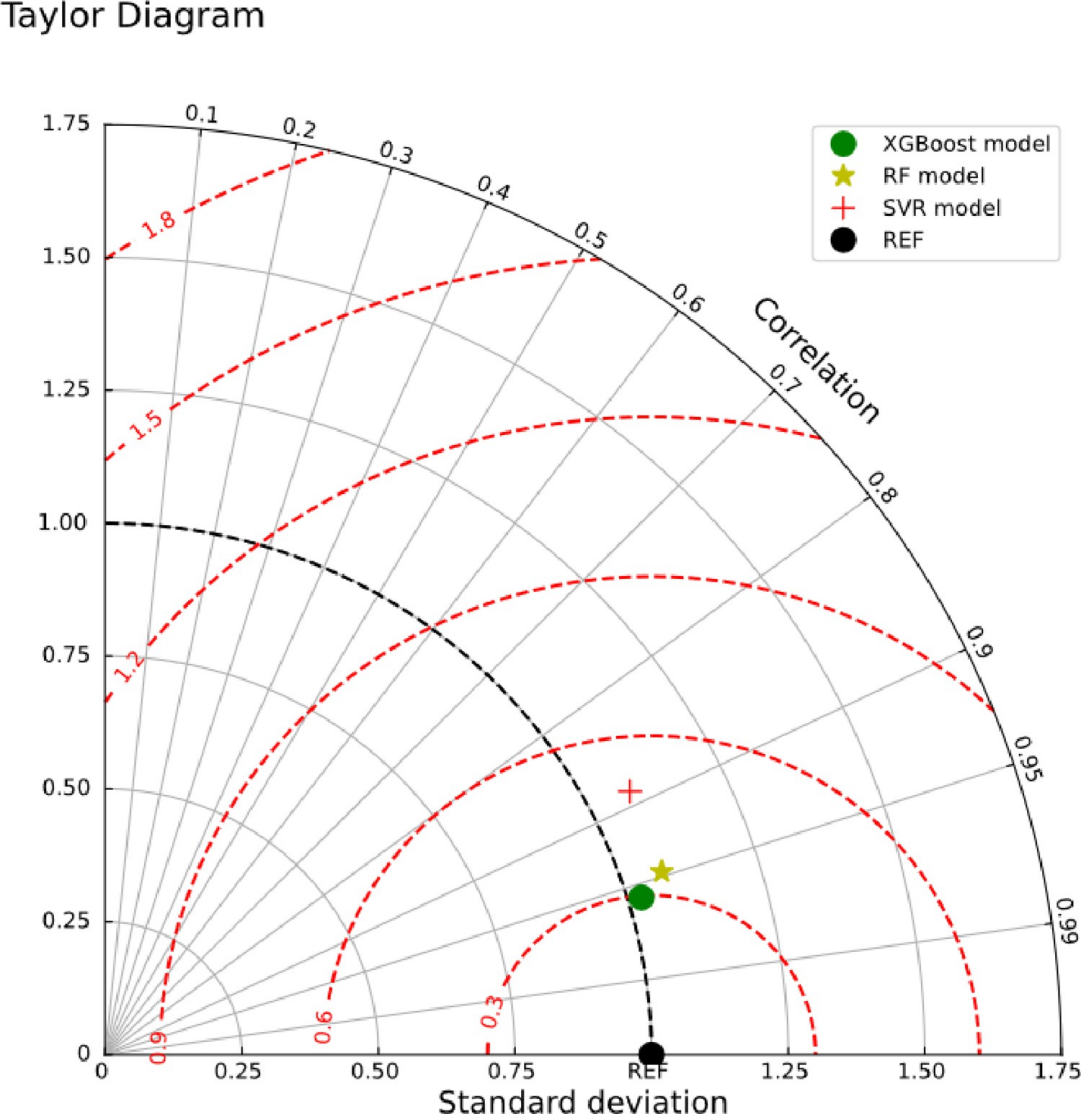
Taylor diagram presentation for the predicted values of compressive strength utilizing machine learning model.

Fig 7 summarizes the general performances of the applied models in the form of the Taylor diagram [43]. This diagram simply expresses three main statistics including the correlation coefficient between the predicted values and measured data (as an angle in the polar plot), RMSE (as a radial distance from the observation point), and the ratio of the standard deviation of the predicted values (as a radial distance from the origin). As can be observed in Fig 7, the XGBoost molel is closer to the REF point in comparison with the other two predictive modelling approaches (RF, and SVR).

## 6. Experimental verification

**Fig 8:**
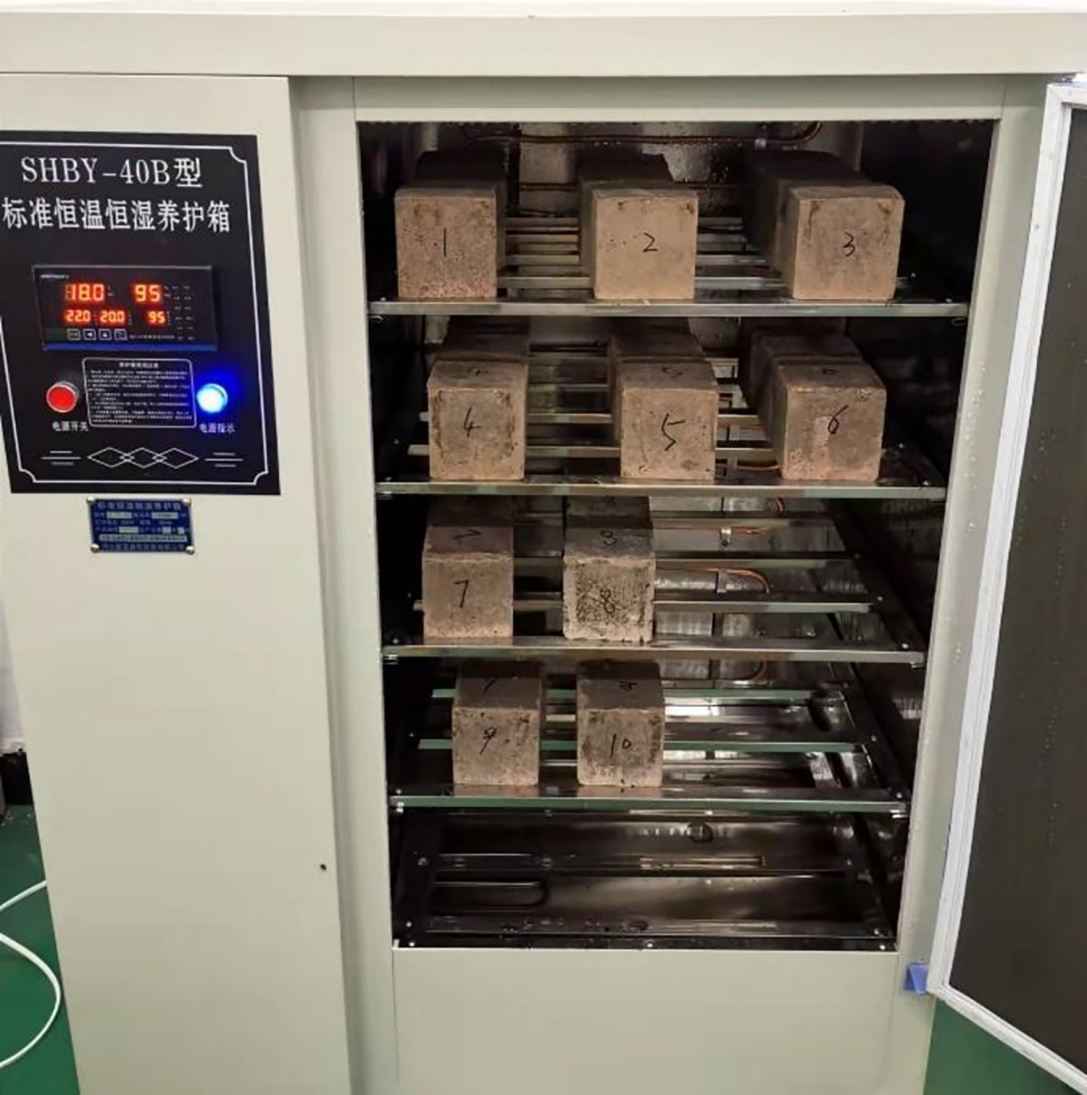
Curing of test block.

In laboratory was configured with a 100×100×100mm concrete test block with the same characteristics as the dataset. The test block is placed in a constant temperature curing box and cured for 28 days at 18 degrees Celsius humidity of 95%, as shown in Fig 8. To prevent the occurrence of accidents such as damage and increase the number of samples to reduce the contingency, 10 groups of proportioning are prepared for this time, and 3 test blocks are made for each proportioning, with a total of 30 test blocks. The concrete compressive strength test was carried out on the test block with the ideal appearance among the three test blocks of each mixing ratio; the concrete mix ratio, predicted strength, and experimental strength was obtained, as shown in Table 5. Finally, the average error rate of 10 combinations of test blocks is calculated, and the average error rate is 3.5%, which is low, and the model’s accuracy is in line with the actual engineering needs [44]. The formula for the average error rate is as follows:

$$e=\frac{\sum_{i=1}^{n}\left(\frac{\left|x^{*}-x\right|}{x^{*}}\right)}{n}\times 100\%$$ (15)

**Table 5:** Actual and predicted compressive strength and error rate of each proportioned concrete test block.

cement (kg/m^3^)	blast furnace slag (kg /m^3^)	fly ash (kg /m^3^)	water (kg /m^3^)	Super plasticizer (kg /m^3^)	coarse aggregate (kg /m^3^)	fine aggregate (kg /m^3^)	age (day)	forecast Compression strength (MPa)	Actual Compression strength (MPa)	error rate
271	38	77	245	9.69	680	1020	28	23.9	25.6	6.6%
277	40	79	245	9.9	677	1016	28	23.92	24.21	1.2%
369	53	106	245	13.19	632	948	28	30.93	33.38	7.3%
380	54	108	245	13	626	940	28	36.43	37.53	2.9%
391	55	112	245	13.97	621	932	28	37.56	38.45	2.3%
403	58	115	245	14.39	615	923	28	40	39.05	2.4%
324	46	92	245	11.59	654	981	28	30.2	27.78	8.7%
341	48	97	245	12.18	646	969	28	29.8	30.36	1.8%
380	54	108	245	13	626	940	28	36.43	36.79	1%
391	55	111	245	13	621	931	28	37.56	37.42	0.3%

In the Eq (15): *e* is the average error rate; x* is the actual value; *x* is the predicted value; *n* is the total number of samples.

## 7. Conclusion

1. After the initial model training of the three machine learning models, the characteristic data are input into the model to predict the compressive strength of concrete. Then, the fitting curves of the actual values and predicted values of the three models are compared, and the data of performance indicators such as R^2^, RMSE and MAE are compared. The results show that XGBoost has better performance in predicting concrete compressive strength. R^2^, RMSE and MAE are 0.95, 0.05 and 0.15, respectively. R^2^ is closer to 1, RMSE and MAE are closer to 0, and the error is smaller, more effective than SVR and RF models.
2. In the laboratory will insert fly ash and ground slag as admixture into concrete test block, using the hydraulic universal testing machine to validate its compressive strength, and use the trained XGBoost model to predict the compressive strength, the results show that the XGBoost models to predict the concrete compressive strength performance good, easy to model, computing speed is fast. The actual average error rate is only 3.5%, which provides an effective method for predicting the compressive strength of concrete with a known mix ratio in advance.
3. By using traditional solid waste fly ash and blast furnace slag as a concrete admixture to make concrete, the concrete finally obtained has high compressive strength and can be used stably for a long time, which meets the requirements of engineering strength. It has guiding significance for engineering applications and realizes the goal of recycling solid waste such as fly ash and slag.
4. The machine learning model is used to predict the compressive strength of concrete. The compressive strength data of concrete made using fly ash, and blast furnace slag as concrete mixing material can be obtained through fewer experiments. Compared with the previous experiments, concrete samples are prepared in large quantities to test their compressive strength. Predicting its compressive strength through a machine learning model saves experimental costs and resources.

## 8. Limitation and future research direction

1. This paper investigates the compressive strength of concrete with fly ash and slag as admixture materials. The mechanical properties, such as tensile and flexural strength, are not investigated. They need to be further explored to establish a complete predictive model for concrete mechanics of solid waste materials such as fly ash and slag.
2. In this paper, the compressive strength of concrete was investigated using the ratio of eight different materials as the input parameters of the prediction model. Still, many factors affect the compressive strength of concrete with solid waste materials such as fly ash and slag. There are constraints among the factors so that subsequent studies can consider as many different factors as possible as input parameters, such as aggregate particle size, each component of solid waste materials ratio, and other factors.
